# 2-Hy­droxy-16-[(*E*)-4-methyl­benzyl­idene]-13-(4-methyl­phen­yl)-12-phenyl-1,11-diaza­penta­cyclo­[12.3.1.0^2,10^.0^3,8^.0^10,14^]octa­deca-3(8),4,6-triene-9,15-dione

**DOI:** 10.1107/S1600536810028357

**Published:** 2010-07-24

**Authors:** Raju Suresh Kumar, Hasnah Osman, Aisyah Saad Abdul Rahim, Jia Hao Goh, Hoong-Kun Fun

**Affiliations:** a School of Chemical Sciences, Universiti Sains Malaysia, 11800 USM, Penang, Malaysia; b School of Pharmaceutical Sciences, Universiti Sains Malaysia, 11800 USM, Penang, Malaysia; c X-ray Crystallography Unit, School of Physics, Universiti Sains Malaysia, 11800 USM, Penang, Malaysia

## Abstract

In the title compound, C_37_H_32_N_2_O_3_, an intra­molecular O—H⋯N hydrogen bond generates a five-membered ring, producing an *S*(5) motif. The piperidone ring adopts a half-chair conformation. The two fused pyrrolidine rings have similar envelope conformations. The interplanar angles between the benzene rings A/B and C/D are 75.68 (7) and 30.22 (6)°, respectively. In the crystal structure, adjacent mol­ecules are inter­connected into chains propagating along the [010] direction *via* inter­molecular C—H⋯O hydrogen bonds. Further stabilization is provided by weak C—H⋯π inter­actions.

## Related literature

For general background to and applications of related structures, see: Ban *et al.* (1974 ▶); De Amici *et al.* (1990 ▶); Howe & Shelton (1990 ▶); Kornet & Thio (1976 ▶); Kozikowski (1984 ▶); Li *et al.* (1996 ▶); Okita & Isobe (1994 ▶); Rosenmond *et al.* (1994 ▶); Southon & Buckingham (1989 ▶). For ring puckering analysis, see: Cremer & Pople (1975 ▶). For graph-set descriptions of hydrogen-bond ring motifs, see: Bernstein *et al.* (1995 ▶). For closely related structures, see: Kumar *et al.* (2010**a* ▶,*b* ▶,c* ▶). For the stability of the temperature controller used in the data collection, see: Cosier & Glazer (1986 ▶).
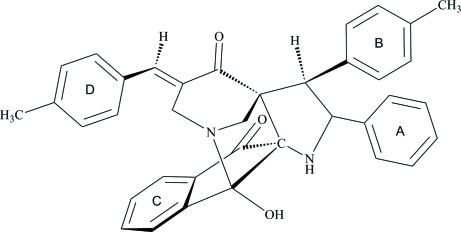


## Experimental

### 

#### Crystal data

C_37_H_32_N_2_O_3_
*M* *_r_* = 552.65
Monoclinic, 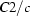

*a* = 42.936 (5) Å
*b* = 7.3759 (9) Å
*c* = 17.990 (2) Å
β = 91.154 (2)°
*V* = 5696.0 (12) Å^3^
*Z* = 8
Mo *K*α radiation
μ = 0.08 mm^−1^
*T* = 100 K
0.48 × 0.15 × 0.12 mm

#### Data collection

Bruker APEXII DUO CCD area-detector diffractometer
Absorption correction: multi-scan (*SADABS*; Bruker, 2009 ▶) *T*_min_ = 0.962, *T*_max_ = 0.990
21929 measured reflections
7946 independent reflections
6021 reflections with *I* > 2σ(*I*)
*R*_int_ = 0.034

#### Refinement

*R*[*F* ^2^ > 2σ(*F* ^2^)] = 0.047
*wR*(*F* ^2^) = 0.139
*S* = 1.04
7946 reflections
389 parameters
H atoms treated by a mixture of independent and constrained refinement
Δρ_max_ = 0.44 e Å^−3^
Δρ_min_ = −0.23 e Å^−3^

### 

Data collection: *APEX2* (Bruker, 2009 ▶); cell refinement: *SAINT* (Bruker, 2009 ▶); data reduction: *SAINT*; program(s) used to solve structure: *SHELXTL* (Sheldrick, 2008 ▶); program(s) used to refine structure: *SHELXTL*; molecular graphics: *SHELXTL*; software used to prepare material for publication: *SHELXTL* and *PLATON* (Spek, 2009 ▶).

## Supplementary Material

Crystal structure: contains datablocks global, I. DOI: 10.1107/S1600536810028357/rz2478sup1.cif

Structure factors: contains datablocks I. DOI: 10.1107/S1600536810028357/rz2478Isup2.hkl

Additional supplementary materials:  crystallographic information; 3D view; checkCIF report

## Figures and Tables

**Table 1:** Hydrogen-bond geometry (Å, °) *Cg*1 and *Cg*2 are the centroids of the C9–C14 and C18–C23 benzene rings, respectively.

*D*—H⋯*A*	*D*—H	H⋯*A*	*D*⋯*A*	*D*—H⋯*A*
O1—H1*O*1⋯N1	0.98 (2)	1.94 (2)	2.6477 (15)	126.7 (18)
C22—H22*A*⋯O1^i^	0.93	2.42	3.2564 (16)	149
C3—H3*A*⋯*Cg*1^ii^	0.93	2.73	3.5620 (18)	150
C37—H37*B*⋯*Cg*2^iii^	0.96	2.61	3.3932 (16)	139

Symmetry codes: (i) 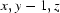; (ii) 
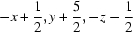
; (iii) 
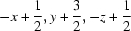
.

**Fig. 1.**
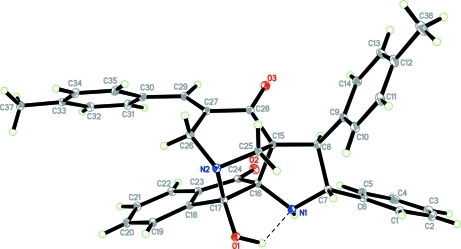
The molecular structure of the title compound, showing 20 % probability displacement ellipsoids for non-H atoms and the atom-numbering scheme. An intramolecular hydrogen bond is shown as dashed line.

**Fig. 2.**
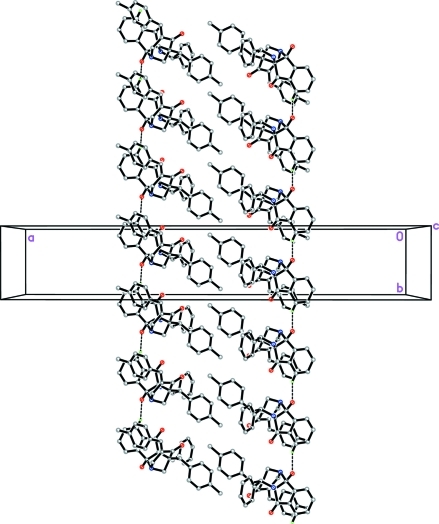
The crystal structure of the title compound, viewed down the c axis, showing infinite chains propagating along the [010] direction. H atoms not involved in intermolecular hydrogen bonds (dashed lines) have been omitted for clarity.

## supplementary crystallographic information

### Comment

The versatility of 1,3-dipolar cycloadditions for the construction of five-membered heterocyclic rings is well established. The reaction of azomethine ylides with alkenes affords pyrrolidines which are present in numerous alkaloids (Southon & Buckingham, 1989) and physiologically active compounds (Li *et al.*, 1996). Spiropyrrolidines have received considerable attention due to its biological activity (Kozikowski, 1984; Howe & Shelton, 1990; De Amici *et al.*, 1990; Ban *et al.*, 1974). They display interesting anti-microbial, anti-tumor and antibiotic properties besides acting as inhibitors of human NK-I receptor activity (Okita & Isobe, 1994; Rosenmond *et al.*, 1994; Kornet & Thio, 1976). The biological significance of the above mentioned heterocycles, prompted us to synthesize the title compound whose crystal structure is presented here.

The molecular structure of the title compound is shown in Fig. 1. An intramolecular O1—H1O1···N1 hydrogen bond (Table 1) forms a five-membered ring, generating an *S*(5) hydrogen bond ring motif (Bernstein *et al.*, 1995). The 4-piperidone ring (N2/C15/C25-C28) adopts a half-chair conformation, with puckering parameters Q = 0.6128 (13) Å, θ = 36.28 (12)° and φ = 58.1 (2)° (Cremer & Pople, 1975). The two fused pyrrolidine rings with atom sequences C7/C8/C15/C16/N1 and C15/C16/C17/N2/C25, adopt similar envelope conformations, with atoms C8 and C25, respectively, as the flap atoms. The puckering parameters are Q = 0.3433 (14) Å, φ = 77.2 (2)° for the C7/C8/C15/C16/N1 pyrrolidine ring and Q = 0.4564 (13) Å, φ = 331.35 (17)° for the C15/C16/C17/N2/C25 pyrrolidine ring. The benzene rings A/B and C/D form interplanar angles of 75.68 (7) and 30.22 (6)°, respectively. The geometric parameters are consistent to those observed in closely related structures (Kumar *et al.*, 2010**a*,*b*,c*).

In the crystal structure, adjacent molecules are interconnected into one-dimensional chains propagating along the [010] direction *via* intermolecular C22—H22A···O1 hydrogen bonds (Fig. 2, Table 1). Further stabilization is provided by weak intermolecular C3—H3A···*Cg*1 and C37—H37B···*Cg*2 interactions involving the C9-C14 (*Cg*1) and C18-C23 (*Cg*2) benzene rings.

### Experimental

A mixture of 3,5-bis[(*E*)-(4-methylphenyl)methylidene]tetrahydro-4(1*H*)-pyridinone (0.100 g, 0.330 mmol), ninhydrin (0.059 g, 0.330 mmol) and phenylglycine (0.050 g, 0.330 mmol) were dissolved in methanol (10 ml) and refluxed for 1 h. After completion of the reaction as evident from TLC, the mixture was poured into water (50 ml). The solid precipitated was filtered and washed with water to afford the product which was recrystallized from ethyl acetate to reveal the title compound as colourless crystals.

### Refinement

Atoms H1N1 and H1O1 were located from difference Fourier map [N1—H1N1 = 0.853 (19) Å and O1—H1O1 = 0.98 (2) Å] and allowed to refine freely. The remaining H atoms were placed in their calculated positions, with C—H = 0.93 – 0.97 Å, and refined using a riding model, with *U*_iso_ = 1.2 or 1.5 *U*_eq_(C). The rotating group model was applied to the methyl groups.

### Crystal data

**Table:**

C_37_H_32_N_2_O_3_	*F*(000) = 2336
*M**_r_* = 552.65	*D*_x_ = 1.289 Mg m^−^^3^
Monoclinic, *C*2/*c*	Mo *K*α radiation, λ = 0.71073 Å
Hall symbol: -C 2yc	Cell parameters from 5678 reflections
*a* = 42.936 (5) Å	θ = 2.4–29.4°
*b* = 7.3759 (9) Å	µ = 0.08 mm^−^^1^
*c* = 17.990 (2) Å	*T* = 100 K
β = 91.154 (2)°	Block, colourless
*V* = 5696.0 (12) Å^3^	0.48 × 0.15 × 0.12 mm
*Z* = 8	

### Data collection

**Table:**

Bruker APEXII DUO CCD area-detector diffractometer	7946 independent reflections
Radiation source: fine-focus sealed tube	6021 reflections with *I* > 2σ(*I*)
graphite	*R*_int_ = 0.034
φ and ω scans	θ_max_ = 29.6°, θ_min_ = 1.9°
Absorption correction: multi-scan (*SADABS*; Bruker, 2009)	*h* = −59→57
*T*_min_ = 0.962, *T*_max_ = 0.990	*k* = −10→10
21929 measured reflections	*l* = −17→24

### Refinement

**Table:**

Refinement on *F*^2^	Primary atom site location: structure-invariant direct methods
Least-squares matrix: full	Secondary atom site location: difference Fourier map
*R*[*F*^2^ > 2σ(*F*^2^)] = 0.047	Hydrogen site location: inferred from neighbouring sites
*wR*(*F*^2^) = 0.139	H atoms treated by a mixture of independent and constrained refinement
*S* = 1.04	*w* = 1/[σ^2^(*F*_o_^2^) + (0.075*P*)^2^ + 2.0745*P*] where *P* = (*F*_o_^2^ + 2*F*_c_^2^)/3
7946 reflections	(Δ/σ)_max_ < 0.001
389 parameters	Δρ_max_ = 0.44 e Å^−^^3^
0 restraints	Δρ_min_ = −0.22 e Å^−^^3^

### Special details

**Table:**

Experimental. The crystal was placed in the cold stream of an Oxford Cryosystems Cobra open-flow nitrogen cryostat (Cosier & Glazer, 1986) operating at 100.0 (1)K.
Geometry. All esds (except the esd in the dihedral angle between two l.s. planes) are estimated using the full covariance matrix. The cell esds are taken into account individually in the estimation of esds in distances, angles and torsion angles; correlations between esds in cell parameters are only used when they are defined by crystal symmetry. An approximate (isotropic) treatment of cell esds is used for estimating esds involving l.s. planes.
Refinement. Refinement of F^2^ against ALL reflections. The weighted R-factor wR and goodness of fit S are based on F^2^, conventional R-factors R are based on F, with F set to zero for negative F^2^. The threshold expression of F^2^ > 2sigma(F^2^) is used only for calculating R-factors(gt) etc. and is not relevant to the choice of reflections for refinement. R-factors based on F^2^ are statistically about twice as large as those based on F, and R- factors based on ALL data will be even larger.

### Fractional atomic coordinates and isotropic or equivalent isotropic displacement parameters (Å^2^)

**Table:**

	*x*	*y*	*z*	*U*_iso_*/*U*_eq_	
O1	0.18627 (2)	1.03210 (12)	0.30204 (5)	0.01996 (19)	
O2	0.13482 (2)	0.48587 (12)	0.29378 (6)	0.0250 (2)	
O3	0.08307 (2)	0.67065 (13)	0.41752 (5)	0.0241 (2)	
N1	0.13953 (3)	0.86274 (16)	0.23346 (6)	0.0199 (2)	
N2	0.15966 (2)	1.00834 (14)	0.41487 (6)	0.0178 (2)	
C1	0.08158 (3)	1.0494 (2)	0.10439 (8)	0.0304 (3)	
H1A	0.0854	1.1698	0.1171	0.036*	
C2	0.06469 (4)	1.0090 (3)	0.03965 (9)	0.0421 (5)	
H2A	0.0569	1.1022	0.0098	0.050*	
C3	0.05942 (4)	0.8310 (3)	0.01968 (9)	0.0442 (5)	
H3A	0.0482	0.8042	−0.0237	0.053*	
C4	0.07088 (4)	0.6919 (3)	0.06415 (9)	0.0379 (4)	
H4A	0.0676	0.5719	0.0502	0.046*	
C5	0.08731 (3)	0.7314 (2)	0.12971 (8)	0.0287 (3)	
H5A	0.0946	0.6377	0.1599	0.034*	
C6	0.09286 (3)	0.9115 (2)	0.15028 (7)	0.0220 (3)	
C7	0.10909 (3)	0.95777 (18)	0.22299 (7)	0.0192 (3)	
H7A	0.1129	1.0888	0.2242	0.023*	
C8	0.08979 (3)	0.90609 (17)	0.29166 (7)	0.0177 (2)	
H8A	0.0808	0.7859	0.2824	0.021*	
C9	0.06333 (3)	1.03429 (18)	0.30922 (7)	0.0200 (3)	
C10	0.06675 (3)	1.22327 (19)	0.30781 (8)	0.0242 (3)	
H10A	0.0857	1.2737	0.2944	0.029*	
C11	0.04219 (3)	1.3357 (2)	0.32621 (9)	0.0287 (3)	
H11A	0.0451	1.4607	0.3255	0.034*	
C12	0.01336 (3)	1.2665 (2)	0.34572 (8)	0.0293 (3)	
C13	0.00988 (3)	1.0786 (2)	0.34639 (8)	0.0291 (3)	
H13A	−0.0092	1.0286	0.3590	0.035*	
C14	0.03436 (3)	0.9650 (2)	0.32861 (8)	0.0238 (3)	
H14A	0.0314	0.8401	0.3296	0.029*	
C15	0.11491 (3)	0.88589 (16)	0.35278 (7)	0.0170 (2)	
C16	0.14402 (3)	0.81013 (16)	0.31136 (7)	0.0168 (2)	
C17	0.17248 (3)	0.90762 (16)	0.35090 (7)	0.0167 (2)	
C18	0.19448 (3)	0.75676 (17)	0.37353 (7)	0.0171 (2)	
C19	0.22436 (3)	0.77126 (18)	0.40446 (7)	0.0198 (3)	
H19A	0.2335	0.8841	0.4125	0.024*	
C20	0.24021 (3)	0.61228 (19)	0.42312 (7)	0.0223 (3)	
H20A	0.2603	0.6194	0.4432	0.027*	
C21	0.22647 (3)	0.44229 (19)	0.41226 (8)	0.0229 (3)	
H21A	0.2372	0.3384	0.4271	0.027*	
C22	0.19708 (3)	0.42651 (17)	0.37970 (8)	0.0213 (3)	
H22A	0.1880	0.3135	0.3715	0.026*	
C23	0.18143 (3)	0.58607 (17)	0.35960 (7)	0.0181 (2)	
C24	0.15120 (3)	0.60572 (17)	0.31985 (7)	0.0183 (2)	
C25	0.12859 (3)	1.06363 (17)	0.38670 (7)	0.0188 (2)	
H25A	0.1302	1.1574	0.3492	0.023*	
H25B	0.1158	1.1079	0.4267	0.023*	
C26	0.15543 (3)	0.89938 (17)	0.48297 (7)	0.0184 (2)	
H26A	0.1486	0.9792	0.5223	0.022*	
H26B	0.1755	0.8498	0.4983	0.022*	
C27	0.13222 (3)	0.74384 (17)	0.47496 (7)	0.0181 (2)	
C28	0.10700 (3)	0.75907 (17)	0.41627 (7)	0.0183 (2)	
C29	0.13492 (3)	0.58452 (18)	0.51089 (7)	0.0201 (3)	
H29A	0.1197	0.4982	0.5001	0.024*	
C30	0.15930 (3)	0.53178 (18)	0.56535 (7)	0.0199 (3)	
C31	0.17147 (3)	0.35579 (18)	0.56138 (8)	0.0231 (3)	
H31A	0.1635	0.2751	0.5261	0.028*	
C32	0.19527 (3)	0.30077 (18)	0.60963 (8)	0.0228 (3)	
H32A	0.2030	0.1834	0.6061	0.027*	
C33	0.20786 (3)	0.41771 (18)	0.66347 (7)	0.0203 (3)	
C34	0.19511 (3)	0.59187 (19)	0.66842 (7)	0.0232 (3)	
H34A	0.2027	0.6713	0.7046	0.028*	
C35	0.17126 (3)	0.64854 (18)	0.62047 (7)	0.0225 (3)	
H35A	0.1632	0.7649	0.6250	0.027*	
C36	−0.01298 (4)	1.3907 (3)	0.36736 (11)	0.0440 (4)	
H36A	−0.0091	1.5106	0.3490	0.066*	
H36B	−0.0143	1.3941	0.4205	0.066*	
H36C	−0.0322	1.3462	0.3463	0.066*	
C37	0.23421 (3)	0.35823 (19)	0.71403 (8)	0.0234 (3)	
H37A	0.2320	0.2315	0.7250	0.035*	
H37B	0.2338	0.4266	0.7594	0.035*	
H37D	0.2537	0.3786	0.6900	0.035*	
H1O1	0.1756 (5)	1.015 (3)	0.2538 (13)	0.056 (6)*	
H1N1	0.1405 (4)	0.767 (3)	0.2071 (10)	0.032 (5)*	

### Atomic displacement parameters (Å^2^)

**Table:**

	*U*^11^	*U*^22^	*U*^33^	*U*^12^	*U*^13^	*U*^23^
O1	0.0211 (5)	0.0179 (4)	0.0209 (5)	−0.0028 (3)	0.0006 (4)	0.0031 (4)
O2	0.0225 (5)	0.0194 (4)	0.0330 (5)	−0.0036 (4)	−0.0018 (4)	−0.0046 (4)
O3	0.0216 (5)	0.0251 (5)	0.0254 (5)	−0.0044 (4)	−0.0012 (4)	0.0033 (4)
N1	0.0183 (5)	0.0239 (6)	0.0177 (5)	0.0019 (4)	0.0002 (4)	−0.0017 (4)
N2	0.0192 (5)	0.0162 (5)	0.0179 (5)	0.0014 (4)	0.0002 (4)	−0.0006 (4)
C1	0.0224 (7)	0.0431 (9)	0.0257 (7)	0.0004 (6)	0.0035 (5)	0.0129 (6)
C2	0.0246 (8)	0.0774 (14)	0.0242 (8)	0.0027 (8)	0.0005 (6)	0.0195 (8)
C3	0.0224 (7)	0.0920 (15)	0.0181 (7)	−0.0063 (8)	−0.0005 (6)	−0.0006 (8)
C4	0.0241 (7)	0.0609 (11)	0.0289 (8)	−0.0092 (7)	0.0039 (6)	−0.0152 (8)
C5	0.0242 (7)	0.0367 (8)	0.0252 (7)	−0.0022 (6)	−0.0008 (5)	−0.0033 (6)
C6	0.0162 (6)	0.0316 (7)	0.0183 (6)	−0.0003 (5)	0.0019 (5)	0.0031 (5)
C7	0.0176 (6)	0.0207 (6)	0.0193 (6)	0.0006 (5)	0.0002 (5)	0.0016 (5)
C8	0.0167 (6)	0.0184 (6)	0.0179 (6)	0.0002 (4)	−0.0002 (4)	0.0005 (5)
C9	0.0180 (6)	0.0239 (6)	0.0179 (6)	0.0028 (5)	−0.0020 (5)	−0.0006 (5)
C10	0.0210 (6)	0.0236 (6)	0.0279 (7)	0.0015 (5)	−0.0027 (5)	0.0018 (5)
C11	0.0270 (7)	0.0250 (7)	0.0338 (8)	0.0057 (6)	−0.0063 (6)	−0.0032 (6)
C12	0.0222 (7)	0.0371 (8)	0.0282 (7)	0.0091 (6)	−0.0058 (6)	−0.0090 (6)
C13	0.0182 (6)	0.0400 (8)	0.0291 (8)	0.0011 (6)	0.0000 (5)	−0.0057 (6)
C14	0.0207 (6)	0.0272 (7)	0.0236 (7)	−0.0004 (5)	−0.0003 (5)	−0.0027 (5)
C15	0.0156 (6)	0.0176 (5)	0.0178 (6)	0.0005 (4)	0.0008 (4)	0.0005 (5)
C16	0.0158 (6)	0.0169 (5)	0.0176 (6)	−0.0004 (4)	−0.0002 (4)	−0.0009 (4)
C17	0.0169 (6)	0.0150 (5)	0.0183 (6)	−0.0017 (4)	0.0004 (4)	−0.0006 (4)
C18	0.0181 (6)	0.0168 (5)	0.0162 (6)	0.0008 (4)	0.0008 (4)	−0.0008 (4)
C19	0.0205 (6)	0.0200 (6)	0.0188 (6)	−0.0008 (5)	−0.0012 (5)	−0.0018 (5)
C20	0.0209 (6)	0.0269 (7)	0.0189 (6)	0.0035 (5)	−0.0027 (5)	−0.0017 (5)
C21	0.0255 (7)	0.0219 (6)	0.0213 (6)	0.0067 (5)	−0.0002 (5)	−0.0002 (5)
C22	0.0237 (6)	0.0160 (6)	0.0242 (7)	0.0014 (5)	0.0022 (5)	−0.0019 (5)
C23	0.0181 (6)	0.0179 (6)	0.0184 (6)	0.0002 (4)	0.0015 (5)	−0.0019 (5)
C24	0.0172 (6)	0.0175 (6)	0.0202 (6)	−0.0003 (4)	0.0020 (5)	−0.0019 (5)
C25	0.0188 (6)	0.0168 (6)	0.0208 (6)	0.0008 (4)	0.0007 (5)	−0.0002 (5)
C26	0.0203 (6)	0.0175 (6)	0.0175 (6)	−0.0006 (5)	0.0003 (5)	−0.0008 (5)
C27	0.0191 (6)	0.0193 (6)	0.0160 (6)	0.0001 (5)	0.0012 (4)	−0.0005 (5)
C28	0.0189 (6)	0.0167 (5)	0.0193 (6)	0.0017 (4)	0.0016 (5)	−0.0013 (5)
C29	0.0202 (6)	0.0211 (6)	0.0191 (6)	−0.0018 (5)	0.0004 (5)	0.0017 (5)
C30	0.0219 (6)	0.0201 (6)	0.0178 (6)	−0.0014 (5)	0.0012 (5)	0.0027 (5)
C31	0.0268 (7)	0.0179 (6)	0.0245 (7)	−0.0031 (5)	−0.0027 (5)	−0.0001 (5)
C32	0.0258 (7)	0.0173 (6)	0.0253 (7)	0.0013 (5)	0.0009 (5)	0.0014 (5)
C33	0.0199 (6)	0.0226 (6)	0.0184 (6)	−0.0004 (5)	0.0024 (5)	0.0030 (5)
C34	0.0281 (7)	0.0242 (6)	0.0172 (6)	0.0006 (5)	−0.0007 (5)	−0.0016 (5)
C35	0.0284 (7)	0.0203 (6)	0.0189 (6)	0.0041 (5)	0.0003 (5)	−0.0005 (5)
C36	0.0301 (8)	0.0494 (10)	0.0524 (11)	0.0153 (7)	−0.0035 (8)	−0.0168 (9)
C37	0.0223 (6)	0.0272 (7)	0.0205 (6)	0.0019 (5)	−0.0003 (5)	0.0018 (5)

### Geometric parameters (Å, °)

**Table:**

O1—C17	1.4095 (15)	C16—C24	1.5458 (17)
O1—H1O1	0.98 (2)	C16—C17	1.5753 (17)
O2—C24	1.2176 (15)	C17—C18	1.5101 (17)
O3—C28	1.2175 (16)	C18—C19	1.3922 (18)
N1—C16	1.4631 (17)	C18—C23	1.3986 (17)
N1—C7	1.4914 (17)	C19—C20	1.3935 (18)
N1—H1N1	0.853 (19)	C19—H19A	0.9300
N2—C25	1.4748 (16)	C20—C21	1.3978 (19)
N2—C26	1.4795 (16)	C20—H20A	0.9300
N2—C17	1.4848 (16)	C21—C22	1.3851 (19)
C1—C6	1.391 (2)	C21—H21A	0.9300
C1—C2	1.392 (2)	C22—C23	1.3992 (17)
C1—H1A	0.9300	C22—H22A	0.9300
C2—C3	1.379 (3)	C23—C24	1.4766 (18)
C2—H2A	0.9300	C25—H25A	0.9700
C3—C4	1.385 (3)	C25—H25B	0.9700
C3—H3A	0.9300	C26—C27	1.5248 (17)
C4—C5	1.393 (2)	C26—H26A	0.9700
C4—H4A	0.9300	C26—H26B	0.9700
C5—C6	1.398 (2)	C27—C29	1.3451 (18)
C5—H5A	0.9300	C27—C28	1.5012 (18)
C6—C7	1.5090 (18)	C29—C30	1.4717 (18)
C7—C8	1.5490 (18)	C29—H29A	0.9300
C7—H7A	0.9800	C30—C31	1.4016 (18)
C8—C9	1.5162 (18)	C30—C35	1.4028 (19)
C8—C15	1.5321 (17)	C31—C32	1.3880 (19)
C8—H8A	0.9800	C31—H31A	0.9300
C9—C14	1.3953 (19)	C32—C33	1.3976 (19)
C9—C10	1.4019 (19)	C32—H32A	0.9300
C10—C11	1.3867 (19)	C33—C34	1.4000 (19)
C10—H10A	0.9300	C33—C37	1.5027 (19)
C11—C12	1.391 (2)	C34—C35	1.3898 (19)
C11—H11A	0.9300	C34—H34A	0.9300
C12—C13	1.394 (2)	C35—H35A	0.9300
C12—C36	1.512 (2)	C36—H36A	0.9600
C13—C14	1.387 (2)	C36—H36B	0.9600
C13—H13A	0.9300	C36—H36C	0.9600
C14—H14A	0.9300	C37—H37A	0.9600
C15—C28	1.5202 (18)	C37—H37B	0.9600
C15—C25	1.5562 (17)	C37—H37D	0.9600
C15—C16	1.5708 (17)		
			
C17—O1—H1O1	105.8 (13)	C19—C18—C23	120.23 (11)
C16—N1—C7	110.12 (10)	C19—C18—C17	128.13 (11)
C16—N1—H1N1	107.8 (12)	C23—C18—C17	111.64 (11)
C7—N1—H1N1	111.8 (12)	C18—C19—C20	118.25 (12)
C25—N2—C26	108.03 (10)	C18—C19—H19A	120.9
C25—N2—C17	102.65 (10)	C20—C19—H19A	120.9
C26—N2—C17	115.06 (10)	C19—C20—C21	121.22 (12)
C6—C1—C2	120.58 (17)	C19—C20—H20A	119.4
C6—C1—H1A	119.7	C21—C20—H20A	119.4
C2—C1—H1A	119.7	C22—C21—C20	120.86 (12)
C3—C2—C1	120.15 (16)	C22—C21—H21A	119.6
C3—C2—H2A	119.9	C20—C21—H21A	119.6
C1—C2—H2A	119.9	C21—C22—C23	117.85 (12)
C2—C3—C4	120.01 (15)	C21—C22—H22A	121.1
C2—C3—H3A	120.0	C23—C22—H22A	121.1
C4—C3—H3A	120.0	C18—C23—C22	121.48 (12)
C3—C4—C5	120.16 (17)	C18—C23—C24	110.10 (11)
C3—C4—H4A	119.9	C22—C23—C24	128.36 (12)
C5—C4—H4A	119.9	O2—C24—C23	127.63 (12)
C4—C5—C6	120.23 (15)	O2—C24—C16	123.90 (12)
C4—C5—H5A	119.9	C23—C24—C16	108.36 (10)
C6—C5—H5A	119.9	N2—C25—C15	103.50 (10)
C1—C6—C5	118.85 (14)	N2—C25—H25A	111.1
C1—C6—C7	119.87 (13)	C15—C25—H25A	111.1
C5—C6—C7	121.18 (12)	N2—C25—H25B	111.1
N1—C7—C6	113.04 (11)	C15—C25—H25B	111.1
N1—C7—C8	105.38 (10)	H25A—C25—H25B	109.0
C6—C7—C8	112.98 (10)	N2—C26—C27	114.92 (10)
N1—C7—H7A	108.4	N2—C26—H26A	108.5
C6—C7—H7A	108.4	C27—C26—H26A	108.5
C8—C7—H7A	108.4	N2—C26—H26B	108.5
C9—C8—C15	115.52 (10)	C27—C26—H26B	108.5
C9—C8—C7	115.34 (11)	H26A—C26—H26B	107.5
C15—C8—C7	102.47 (10)	C29—C27—C28	117.22 (11)
C9—C8—H8A	107.7	C29—C27—C26	124.19 (12)
C15—C8—H8A	107.7	C28—C27—C26	118.11 (11)
C7—C8—H8A	107.7	O3—C28—C27	122.95 (12)
C14—C9—C10	117.58 (12)	O3—C28—C15	123.02 (12)
C14—C9—C8	119.93 (12)	C27—C28—C15	113.92 (11)
C10—C9—C8	122.49 (12)	C27—C29—C30	127.18 (12)
C11—C10—C9	120.66 (13)	C27—C29—H29A	116.4
C11—C10—H10A	119.7	C30—C29—H29A	116.4
C9—C10—H10A	119.7	C31—C30—C35	118.24 (12)
C10—C11—C12	121.71 (14)	C31—C30—C29	118.15 (12)
C10—C11—H11A	119.1	C35—C30—C29	123.61 (12)
C12—C11—H11A	119.1	C32—C31—C30	120.62 (13)
C11—C12—C13	117.58 (13)	C32—C31—H31A	119.7
C11—C12—C36	121.11 (15)	C30—C31—H31A	119.7
C13—C12—C36	121.29 (15)	C31—C32—C33	121.50 (12)
C14—C13—C12	121.15 (14)	C31—C32—H32A	119.2
C14—C13—H13A	119.4	C33—C32—H32A	119.2
C12—C13—H13A	119.4	C32—C33—C34	117.64 (12)
C13—C14—C9	121.31 (14)	C32—C33—C37	121.02 (12)
C13—C14—H14A	119.3	C34—C33—C37	121.34 (12)
C9—C14—H14A	119.3	C35—C34—C33	121.39 (13)
C28—C15—C8	115.74 (10)	C35—C34—H34A	119.3
C28—C15—C25	108.19 (10)	C33—C34—H34A	119.3
C8—C15—C25	117.02 (10)	C34—C35—C30	120.57 (12)
C28—C15—C16	109.24 (10)	C34—C35—H35A	119.7
C8—C15—C16	104.45 (10)	C30—C35—H35A	119.7
C25—C15—C16	100.84 (9)	C12—C36—H36A	109.5
N1—C16—C24	112.06 (10)	C12—C36—H36B	109.5
N1—C16—C15	105.61 (10)	H36A—C36—H36B	109.5
C24—C16—C15	117.32 (10)	C12—C36—H36C	109.5
N1—C16—C17	113.43 (10)	H36A—C36—H36C	109.5
C24—C16—C17	104.44 (10)	H36B—C36—H36C	109.5
C15—C16—C17	103.93 (9)	C33—C37—H37A	109.5
O1—C17—N2	108.92 (10)	C33—C37—H37B	109.5
O1—C17—C18	112.33 (10)	H37A—C37—H37B	109.5
N2—C17—C18	113.54 (10)	C33—C37—H37D	109.5
O1—C17—C16	110.32 (10)	H37A—C37—H37D	109.5
N2—C17—C16	106.38 (10)	H37B—C37—H37D	109.5
C18—C17—C16	105.11 (10)		
			
C6—C1—C2—C3	−1.1 (2)	C24—C16—C17—C18	4.01 (12)
C1—C2—C3—C4	0.2 (2)	C15—C16—C17—C18	127.53 (10)
C2—C3—C4—C5	1.0 (2)	O1—C17—C18—C19	53.74 (17)
C3—C4—C5—C6	−1.2 (2)	N2—C17—C18—C19	−70.41 (16)
C2—C1—C6—C5	0.9 (2)	C16—C17—C18—C19	173.73 (12)
C2—C1—C6—C7	−175.58 (13)	O1—C17—C18—C23	−126.08 (11)
C4—C5—C6—C1	0.3 (2)	N2—C17—C18—C23	109.78 (12)
C4—C5—C6—C7	176.70 (13)	C16—C17—C18—C23	−6.09 (14)
C16—N1—C7—C6	−142.49 (11)	C23—C18—C19—C20	−2.07 (19)
C16—N1—C7—C8	−18.64 (13)	C17—C18—C19—C20	178.13 (12)
C1—C6—C7—N1	−130.42 (13)	C18—C19—C20—C21	−0.9 (2)
C5—C6—C7—N1	53.20 (17)	C19—C20—C21—C22	2.7 (2)
C1—C6—C7—C8	110.02 (14)	C20—C21—C22—C23	−1.3 (2)
C5—C6—C7—C8	−66.36 (16)	C19—C18—C23—C22	3.43 (19)
N1—C7—C8—C9	158.63 (11)	C17—C18—C23—C22	−176.73 (12)
C6—C7—C8—C9	−77.49 (14)	C19—C18—C23—C24	−174.12 (11)
N1—C7—C8—C15	32.27 (12)	C17—C18—C23—C24	5.71 (15)
C6—C7—C8—C15	156.15 (11)	C21—C22—C23—C18	−1.68 (19)
C15—C8—C9—C14	−103.93 (14)	C21—C22—C23—C24	175.39 (13)
C7—C8—C9—C14	136.68 (13)	C18—C23—C24—O2	173.47 (13)
C15—C8—C9—C10	75.22 (16)	C22—C23—C24—O2	−3.9 (2)
C7—C8—C9—C10	−44.16 (17)	C18—C23—C24—C16	−2.83 (14)
C14—C9—C10—C11	0.9 (2)	C22—C23—C24—C16	179.82 (13)
C8—C9—C10—C11	−178.22 (12)	N1—C16—C24—O2	−54.22 (17)
C9—C10—C11—C12	−0.8 (2)	C15—C16—C24—O2	68.20 (17)
C10—C11—C12—C13	0.2 (2)	C17—C16—C24—O2	−177.41 (12)
C10—C11—C12—C36	178.59 (15)	N1—C16—C24—C23	122.25 (11)
C11—C12—C13—C14	0.3 (2)	C15—C16—C24—C23	−115.33 (12)
C36—C12—C13—C14	−178.10 (14)	C17—C16—C24—C23	−0.94 (13)
C12—C13—C14—C9	−0.2 (2)	C26—N2—C25—C15	−73.87 (11)
C10—C9—C14—C13	−0.5 (2)	C17—N2—C25—C15	48.11 (11)
C8—C9—C14—C13	178.72 (13)	C28—C15—C25—N2	71.91 (12)
C9—C8—C15—C28	80.11 (14)	C8—C15—C25—N2	−155.19 (10)
C7—C8—C15—C28	−153.65 (10)	C16—C15—C25—N2	−42.68 (11)
C9—C8—C15—C25	−49.30 (15)	C25—N2—C26—C27	51.24 (13)
C7—C8—C15—C25	76.94 (12)	C17—N2—C26—C27	−62.74 (14)
C9—C8—C15—C16	−159.75 (10)	N2—C26—C27—C29	146.41 (12)
C7—C8—C15—C16	−33.51 (12)	N2—C26—C27—C28	−25.38 (16)
C7—N1—C16—C24	126.20 (11)	C29—C27—C28—O3	27.66 (18)
C7—N1—C16—C15	−2.66 (13)	C26—C27—C28—O3	−159.97 (12)
C7—N1—C16—C17	−115.84 (11)	C29—C27—C28—C15	−148.73 (12)
C28—C15—C16—N1	147.51 (10)	C26—C27—C28—C15	23.64 (15)
C8—C15—C16—N1	23.11 (12)	C8—C15—C28—O3	3.79 (18)
C25—C15—C16—N1	−98.70 (11)	C25—C15—C28—O3	137.36 (12)
C28—C15—C16—C24	21.84 (15)	C16—C15—C28—O3	−113.72 (13)
C8—C15—C16—C24	−102.57 (12)	C8—C15—C28—C27	−179.82 (10)
C25—C15—C16—C24	135.63 (11)	C25—C15—C28—C27	−46.25 (13)
C28—C15—C16—C17	−92.83 (11)	C16—C15—C28—C27	62.67 (13)
C8—C15—C16—C17	142.76 (10)	C28—C27—C29—C30	174.18 (12)
C25—C15—C16—C17	20.96 (11)	C26—C27—C29—C30	2.3 (2)
C25—N2—C17—O1	85.20 (11)	C27—C29—C30—C31	−138.76 (14)
C26—N2—C17—O1	−157.73 (10)	C27—C29—C30—C35	40.5 (2)
C25—N2—C17—C18	−148.83 (10)	C35—C30—C31—C32	−1.6 (2)
C26—N2—C17—C18	−31.75 (14)	C29—C30—C31—C32	177.71 (12)
C25—N2—C17—C16	−33.71 (12)	C30—C31—C32—C33	0.0 (2)
C26—N2—C17—C16	83.36 (12)	C31—C32—C33—C34	1.5 (2)
N1—C16—C17—O1	3.03 (14)	C31—C32—C33—C37	−178.33 (13)
C24—C16—C17—O1	125.32 (10)	C32—C33—C34—C35	−1.5 (2)
C15—C16—C17—O1	−111.16 (10)	C37—C33—C34—C35	178.38 (13)
N1—C16—C17—N2	121.02 (11)	C33—C34—C35—C30	−0.1 (2)
C24—C16—C17—N2	−116.69 (10)	C31—C30—C35—C34	1.7 (2)
C15—C16—C17—N2	6.83 (12)	C29—C30—C35—C34	−177.62 (13)
N1—C16—C17—C18	−118.28 (11)		

### Hydrogen-bond geometry (Å, °)

**Table:**

Cg1 and Cg2 are the centroids of the C9–C14 and C18–C23 benzene rings, respectively.

**Table:**

*D*—H···*A*	*D*—H	H···*A*	*D*···*A*	*D*—H···*A*
O1—H1O1···N1	0.98 (2)	1.94 (2)	2.6477 (15)	126.7 (18)
C22—H22A···O1^i^	0.93	2.42	3.2564 (16)	149
C3—H3A···Cg1^ii^	0.93	2.73	3.5620 (18)	150
C37—H37B···Cg2^iii^	0.96	2.61	3.3932 (16)	139

Symmetry codes: (i) *x*, *y*−1, *z*; (ii) −*x*+1/2, *y*+5/2, −*z*−1/2; (iii) −*x*+1/2, *y*+3/2, −*z*+1/2.
